# Esophageal cancer and bacterial part of gut microbiota – A multidisciplinary point of view

**DOI:** 10.3389/fcimb.2022.1057668

**Published:** 2022-11-16

**Authors:** Damian Muszyński, Anna Kudra, Bartosz Kamil Sobocki, Marcin Folwarski, Ermanno Vitale, Veronica Filetti, Wojciech Dudzic, Karolina Kaźmierczak-Siedlecka, Karol Połom

**Affiliations:** ^1^ Scientific Circle 4.0 associated with Department of Surgical Oncology, Medical University of Gdansk, Gdansk, Poland; ^2^ Scientific Circle of Oncology and Radiotherapy, Medical University of Gdansk, Gdansk, Poland; ^3^ Department of Clinical Nutrition and Dietetics, Medical University of Gdansk, Gdansk, Poland; ^4^ Department of Clinical and Experimental Medicine, University of Catania, Occupational Medicine, Catania, Italy; ^5^ Department of General and Gastrointestinal Surgery and Nutrition, Copernicus Hospital Gdansk, Gdansk, Poland; ^6^ Department of Surgical Oncology, Medical University of Gdansk, Gdansk, Poland

**Keywords:** esophageal cancer, esophageal microbiome, gut microbiome, oral microbiome, 16S rRNA gene sequencing

## Abstract

There is an urgent need to search for new screening methods that allow early detection of esophageal cancer and thus achieve better clinical outcomes. Nowadays, it is known that the esophagus is not a sterile part of the gastrointestinal tract. It is colonized with various microorganisms therefore a “healthy” esophageal microbiome exists. The dysbiotic changes of esophageal microbiome can lead to the development of esophageal diseases including esophageal cancer. There is a strong consensus in the literature that the intestinal microbiome may be involved in esophageal carcinogenesis. Recently, emphasis has also been placed on the relationship between the oral microbiome and the occurrence of esophageal cancer. According to recent studies, some of the bacteria present in the oral cavity, such as *Tannerella forsythia*, *Streptococcus anginosus*, *Aggregatibacter actinomycetemcomitans*, *Porphyromonas gingivalis*, and *Fusobacterium nucleatum* may contribute to the development of this cancer. Moreover, the oral microbiome of patients with esophageal cancer differs significantly from that of healthy individuals. This opens new insights into the search for a microbiome-associated marker for early identification of patients at high risk for developing this cancer.

## Introduction

**Figure 1:**
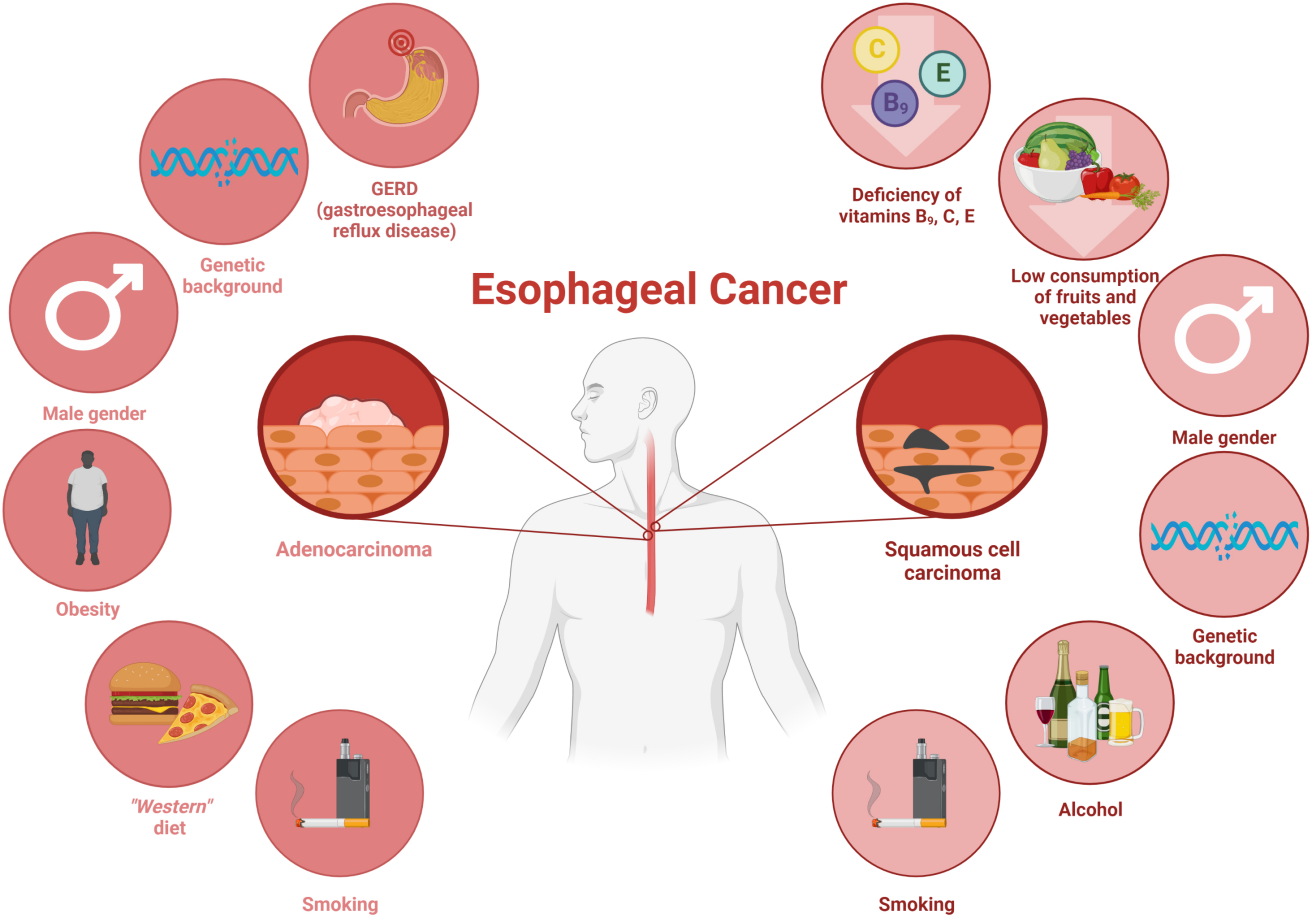
The main risk factors contributing to the development of esophageal cancers: adenocarcinoma and squamous cell carcinoma. Own elaboration based on the literature (Parent et al., 2000; Domper Arnal et al., 2015; Clin et al., 2017; Coleman et al., 2018; Huang and Yu, 2018). This figure was created with *Biorender.com*.

For years, esophageal cancer has been considered as one of the most common cancers worldwide with a reported 604,100 new cases in 2020 (Uhlenhopp et al., 2020; Sung et al., 2021). A common symptom of esophageal cancer is dysphagia (Yuen et al., 2019), which leads to low amount of food intake and consequently contributes to the development of disease-related malnutrition. According to some data, an estimated 79% of these patients are malnourished (Jordan et al., 2018). The etiology of esophageal cancer is complicated and involves several factors, which are shown in **Figure 1** (Huang and Yu, 2018; Uhlenhopp et al., 2020).

As it was mentioned above, there are two types of esophageal cancer (**Figure 1**), namely esophageal squamous cell carcinoma (ESCC, also called OSCC) and esophageal adenocarcinoma (OAC) (Smyth et al., 2017; Short et al., 2017). ESCC is common in East Asia, East Africa, Southern Africa, and Southern Europe, while OAC is more common in developed countries (Huang and Yu, 2018). Over the past four decades, OAC has been found to dominate well ahead of OSCC in terms of incidence (Smyth et al., 2017). The development of esophageal cancer depends on the types of esophageal cancer, due to the fact that they differ in both their biological and anatomical aspects (Smyth et al., 2017). OSCC has similar features to squamous cell carcinoma of the head or neck (Huang and Yu, 2018). In contrast, its precursor in the form of Barrett’s esophagus plays a key role in the development of OAC (Huang and Yu, 2018).

Overall, the above risk factors for esophageal cancer are well described in the literature in contrast to the gut microbiota which, may also play a role in carcinogenesis according to recent published studies (Chen C et al., 2021; Zhou et al., 2021). Therefore, in this review, we briefly discussed the role of the gut microbiota in esophageal carcinogenesis and its alterations in esophageal cancer patients. Then, the relationship between oral microbiota and esophageal cancer development is reviewed based on the recent studies in this field.

## “Healthy” esophageal microbiome

**Figure 2:**
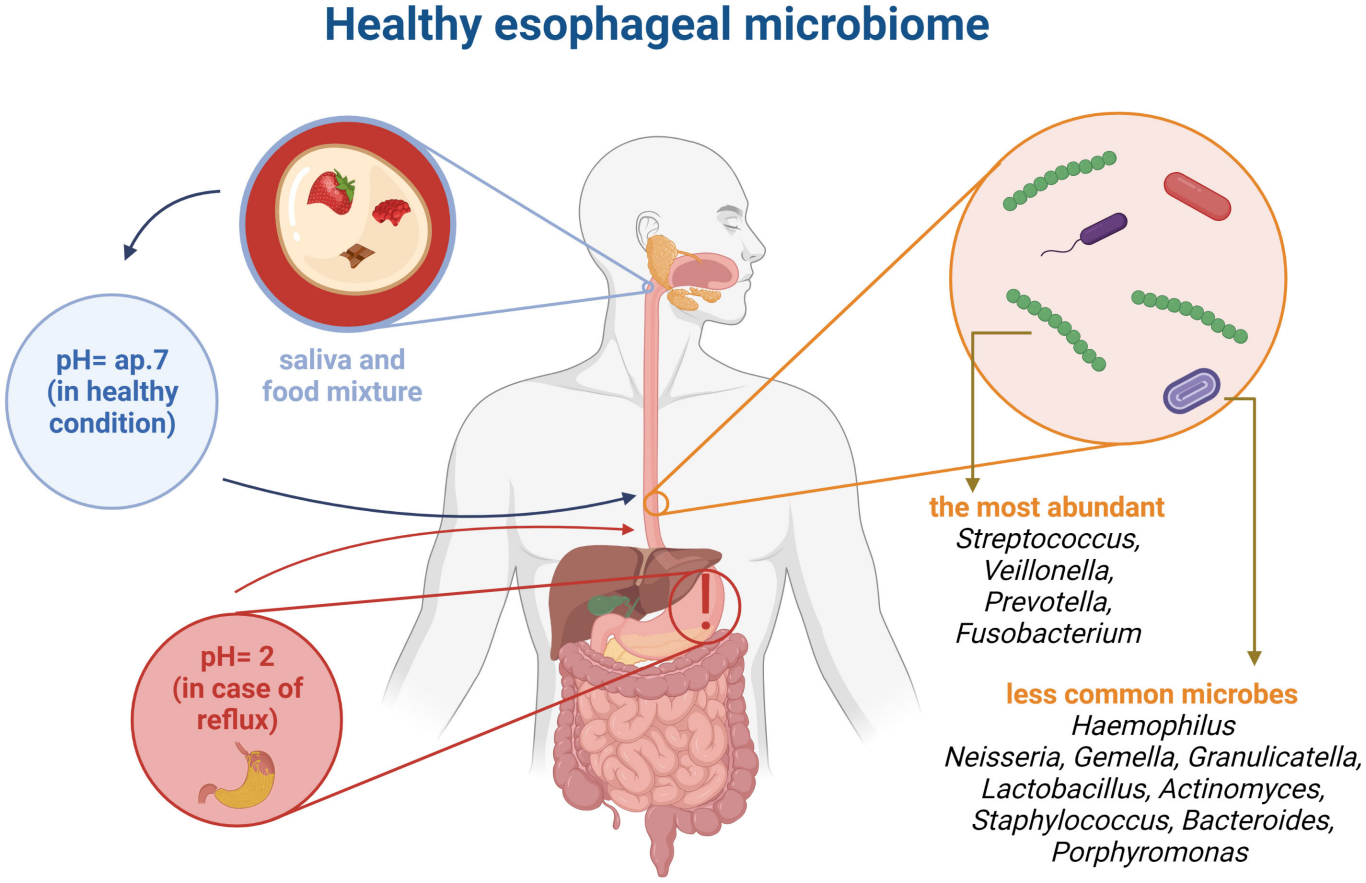
Comparison pointer value pH in healthy organism and with a reflux. Healthy esophageal microbiome based on literature (Di Pilato et al., 2016; Corning et al., 2018; Lv et al., 2019; Laserna-Mendieta et al., 2021). This figure was created using *Biorender.com*.

The esophageal microbiome is not well understood, however it is known that the esophagus is not a sterile part of the gastrointestinal tract (Laserna-Mendieta et al., 2021). Rapid food passage is observed in the esophagus, which probably limits the presence of microbes. Nevertheless, the pH in healthy individuals is quite stable (around 7) providing a good condition for a variety of microbes (Di Pilato et al., 2016). Analysis of the microbiome revealed that the esophagus is resided with some microorganisms (**Figure 2**). Notably, upper, middle as well as lower part of the esophagus have a similar composition of the microbiome. Overall, the esophageal microbiome consists of 6 phyla, such as *Firmicutes*, *Bacteroides*, *Actinobacteria*, *Proteobacteria*, *Fusobacteria*, and *TM7* (Lv et al., 2019). Among the Gram-positive bacteria a diverse microbial population is observed. In particular, the Streptococcus genus is most abundant in the esophagus of healthy individuals (Corning et al., 2018). In addition, *Veillonella* and *Prevotella* also occur in the esophagus (Laserna-Mendieta et al., 2021). In esophageal diseases the microbiome is altered (Lv et al., 2019). The imbalanced changes of esophageal microbiome can be used as a marker for the detection of esophageal diseases (Okereke et al., 2019).

## Gut microbiota and esophageal carcinogenesis

In recent years, new studies have developed our knowledge of the relationship between alterations in the gut microbiota and esophageal carcinogenesis. It has been suggested that this relationship may be crucial in both tumor formation and development (Zhou et al., 2021). Previous studies have confirmed that viruses such as human papillomavirus (HPV) and Epstein-Barr virus, as well as alterations in intestinal bacteria may be involved in esophageal carcinogenesis (Meng et al., 2018). A close association has been noted- high rates of HPV are found in areas that also have high rates of ESCC (Xu et al., 2015). Carcinogenesis can be stimulated by the microbiota in several ways. It has an impact on the immune response. Using a mouse model of Barret’s esophagus, Münch et al. showed that changes in the gut microbiota induced by a high-fat diet led to increased levels of proinflammatory cytokines and immune cells and subsequently to a pro-tumor immune phenotype. Importantly, this study demonstrates the necessary role of the gut microbiota in the transmission of dietary influences *via* inflammatory mechanisms (Münch et al., 2019). A similar mechanism involving the gut microbiota has also been described for a high-fructose diet (Proaño-Vasco et al., 2021). In addition, the study by Lei et al. proved that an adequate composition of the microbiota (in this case, through *Saccharomyces boulardii* supplementation) prevents unfavorable immune responses (Li B et al., 2022). Another study by Wu et al. showed that the gut microbiota plays a mainly protective role and that its perturbations and the decrease in its level lead to an increased inflammatory response and a shortened survival time. In light of these studies, shaping the composition of the gut microbiota may be a useful approach in order to achieve an effective and specific antitumor immune response. The very important part of treatment is adequate supplementation. For instance, riboflavin deficiency leads to oxidative DNA damage and DNA double-strand breaks (Pan et al., 2020). For this reason, new compounds that should be administered to patients with from esophageal cancer need to be better characterized and discovered.

Another potential mechanism linking the microbiota to esophageal carcinogenesis is overexpression of inducible nitric oxide synthase (iNOS) (Ledda et al., 2019; Gillespie et al., 2021). Park et al. indicated that Gram-negative bacteria expressing lipopolysaccharide could increase iNOS expression and thereby decrease esophageal sphincter relaxation, leading to GERD associated with an increased risk of esophageal cancer (Park et al., 2002). The Warburg effect is one of the best known mechanisms involved in carcinogenesis. Studies by Zhou et al. and Deshpande et al. found that lactate-producing bacterial species are significantly increased in Barrett’s esophagus, gastroesophageal reflux disease (GERD), or esophageal adenocarcinoma. Lactate produced by bacteria may be one of the factors that actively support the transition to anaerobic metabolism and stimulate the growth of esophageal cancer (Deshpande et al., 2018; Zhou et al., 2020). The gut microbiota may influence OAC by upregulating of COX-2 and activating Toll-like receptors (Grover et al., 2021). Some driver bacteria can also produce genotoxins that can stimulate cell proliferation and mutation (Sheflin et al., 2014).

The gut microbiota can also secrete some metabolites. One of the examples is polyamines, which promote tumour progression. Another substance of interest is butyrate which generally has tumour-inhibitory properties; however, at low concentrations, it can also stimulate cancer progression (Buda et al., 2003; Clarke et al., 2008; Donohoe et al., 2012; Belcheva et al., 2014; Donohoe et al., 2014).

Although some evidence has now been assembled, the relationship between the gut microbiota and esophageal adenocarcinoma appears to be poorly studied. Future studies should focus on finding possible link mechanisms.

## Dysbiotic alterations of the gut microbiota in patients with esophageal cancer

The association between the gut microbiota and gut microbiota-derived metabolites and many diseases, including cancers, has been studied many times in *in vitro* and *in vivo* studies (Mohseni and Fu, 2020). The gut microbiome was investigated in study by Deng et al. involving 23 patients with esophageal cancer and 23 matched healthy individuals (Deng et al., 2021). The gut microbiome was analysed from fresh stool samples by 16S rRNA gene sequencing. Considering the strain, patients with esophageal cancer were found to have significantly higher levels of Firmicutes and Actinobacteria and lower levels of Bacteroidetes compared to healthy individuals. The authors have reported that the abundance of bacteria, producing short-chain fatty acids (SCFAs) is reduced in patients with esophageal cancer while the amount of lipopolysaccharide-producing bacteria is increased (Deng et al., 2021). It should be emphasized that SCFAs pool plays an important role as it has anti-inflammatory effects and increases the integrity of the intestinal barrier, among others. It is also worth noting that butyrate itself is produced by anaerobes in the distal parts of the digestive system, which may indicate its important role in the development of neoplasms in this system, including in the case of esophageal cancer (Guan et al., 2021). Similarly, in another study in ESCC patients (n=18), it was observed that the amount of *Bacteroidetes*, *Fusobacteria* and *Spirochaetes* is decreased (Yang et al., 2021). Additionally, the diversity of intestinal microbiota is also reduced in these patients.

Other studies showed that there is a relationship between intestinal microbiome and esophageal cancer. Analysis of the feces of patients with esophageal cancer (n=40) in comparison with the feces of healthy peoples (n=147) showed that the microbiome of the two groups is different. In this study, the fecal microbiota was additionally analysed in gastric cancer (n=46) and colorectal cancer (n=44). The differences between cancer patients and control subjects were related to the number of *Bacteroides fragilis*, *Escherichia coli*, *Akkermansia muciniphila*, *Clostridium hathewayi* and *Alistipes finegoldii* in the faeces, while in cancer patients their amount was increased (Li N et al., 2021). In the case of the control group, the increased number of bacteria was especially *Faecalibacterium prausnitzii*, *Roseburia faecis*, *Clostridium clostridioforme*, and also *Bifidobacterium adolescent* (Li N et al., 2021).

High-fat diet (HFD) negatively affects the gut microbiome and the bile acid composition. The changes in bile acids composition induced by HFD may contribute to the development of Barrett’s esophagus and esophageal cancer, as shown in a study in mice (Tong et al., 2021). Overall, obesity is related to higher risk of conditions/disorders, such GERD, Barrett’s esophagus as well as OAC (Lagergren, 2011). Distribution of fat in abdominal part plays a significant role in this context (Lagergren, 2011). It should be also mentioned that OAC, which is associated with obesity, may have a different carcinogenic pathway (Schlottmann et al., 2020).

## Alterations in the oral microbiota

Oral microbiota imbalance can be associated with the development of digestive cancer. Recently in 2021, a systematic review found that there is a difference in the composition of the oral microbiota between patients with digestive cancer and controls subjects (Reitano et al., 2021). Therefore, it is suggested that the oral microbiome may influence the occurrence of esophageal cancer (Peters et al., 2017). Recently, a study by Kawasaki et al. investigated, the relationship between the oral microbiota and esophageal cancer (Kawasaki et al., 2021). In this study, 61 patients with esophageal cancer and 62 matched individuals (without cancer) participated. Samples of both unstimulated saliva and subgingival plaque were collected for analysis of the oral microbiome. The results of this study suggest that bacteria such as *Tannerella forsythia* and *Streptococcus anginosus* (from dental plaque) and *Aggregatibacter actinomycetemcomitans* (from unstimulated saliva) may be associated with higher risk of esophageal cancer (Kawasaki et al., 2021). Notably, virulence factors of *A. actinomycetemcomitans* include leukotoxin and cytotoxic distension toxin. There are seven different serotypes of this bacterium, i.e., serotypes from a to g. Especially, *A. actinomycetemcomitans* serotype b causes an aggressive form of periodontitis. An important virulence factor could be a genomic cagE, which is also a kind of marker (Johansson et al., 2019). The study by Peters et al. also found that *Tannerella forsythia* was associated with a higher risk of EAC whereas *Porphyromonas gingivalis* was associated with a higher risk of ESCC (Peters et al., 2017). In addition, the authors reported that lower levels of *Neisseira* as well as *Streptococcus pneumoniae* may be associated with a lower risk of EAC. The oral microbiome was analysed by 16S rRNA gene sequencing (Peters et al., 2017). *T. forsythia* is known to be a periodontal pathogen. Its virulence is possible thanks to the O-glycan structures, present in the S-layer of this bacterium, which probably play a crucial role in the development of infection (Chinthamani et al., 2021).

*Fusobacterium nucleatum* is known to cause periodontal disease, but may also be associated with tumour development (Yamamura et al., 2016). Rapid disease development is possible thanks to specific virulence factors of *F. nucleatum*, such as adhesin, which allows strong adhesion to host cells (Han, 2015). Recently, it was shown that tissues from esophageal cancer contained significantly more *F. nucleatum* DNA compared with normal esophageal mucosa (p = 0.021). The analysis was performed by qPCR. It has also been reported that *F. nucleatum* is associated with shorter survival in these tissues of esophageal cancer (Yamamura et al., 2016). Another pathogen that may influence carcinogenesis is *P. gingivalis*, which mediates cell transformation in many cancers (Sobocki et al., 2022). Previously, it has been confirmed that the presence of *P. gingivalis* in the esophageal mucosa is a negative prognostic factor that promotes theprognosis of esophageal cancer (Gao et al., 2016). Therefore, eradication of *P. gingivalis* may be considered as a potential treatment option. However, some studies have attempted to investigate the potential mechanism of action. Cell line - based experiments by Liang et al. indicated the role of the miR-194/GRHL3/PTEN/Akt axis. In the group of patients with *P. gingivalis* infection, there were significant differences such as: up-regulation of miR-194 and Akt, down -regulation of GRLH3 (direct target of miR-194) and PTEN compared to patients with negative status. Together, these changes enhanced the pro-proliferative and pro-migratory phenotype of esophageal tumour (Liang et al., 2020). On the other hand, Chen et al. pointed out the mechanism based on the increased production of IL-6 induced by *P. gingivalis* followed by promoted epithelial-mesenhymal transition and recruitment of myeloid – derived suppressor cells (Chen MF et al., 2021). The review by Malinowski et al. indicated other potential mechanisms such as pro-inflammatory IL-1β cytokine production and secretion of gingipain K by *P. gingivalis* which causes degradation of immunoglobulins and the complement system. In addition, infection was associated with significant upregulation of MMP-2 and GLUT transporters (Malinowski et al., 2019). However, these hypotheses should be carefully validated in the future.

Apart from the effects of the above mentioned pathogens, other experiments showed different possibilities of the influence of the oral microbiota. A study on about oral microbiota in patients with esophageal cancer was conducted by Hezi Li et al. (Li H et al., 2021). In this study, microbiota analysis was performed by sequencing the 16S rRNA of V3-V4 gene regions. A variety of microbes were examined and the results showed differences in the esophageal microbiota between healthy individuals and patients with esophageal cancer. At the phylum level, patients with OSCC had a reduced amount of *Proteobacteria* (17.0% vs 20.1% in healthy controls), however slightly higher levels of *Bacteroidetes* (25.3% vs 24.9%) and *Firmicutes* (34% vs 31.1%). At the genus level, the difference between OSCC patients and healthy control subjects is seen in *Streptococcus* (17.3% vs 14.5%), *Prevotella* (8.6% vs 8.5%) and *Neisseria* (8.1% vs 10.7%) (Li H et al., 2021).

In another study, Liu et al. investigated the association between the oral microbiome and the risk of malignant esophageal lesions (Liu et al., 2020). The microbiome was studied by sequencing 16s RNA genes. The results suggest that the oral microbiome, its composition, and its content play an important role the esophageal cancer, and thus may be a biomarker that can be considered in early detection. A similar study in a similar region of the world was performed on 39 patients with esophageal cancer and 51 volunteers as a control group (Zhao et al., 2020). The oral microbiome was analysed by 16S rDNA gene sequencing. The study also revealed differences in the oral microbiome between healthy individuals and patients with esophageal cancer. Notably, in the case of patients with esophageal cancer, increased numbers of *Firmicutes*, *Negativicutes*, *Selenomonadales*, *Prevotellaceae*, *Prevotella*, and *Veillonellaceae* were found in patients with esophageal cancer. The percentage of taxa, such as Proteobacteria, *Betaproteobacteria*, *Neisseriales*, *Neisseriaceae*, and *Neisseria* was reduced. In conclusion the significant difference in the oral microbiome between healthy individuals and those with esophageal cancer suggests that the oral microbiome may be used as a biomarker for the prediction of esophageal cancer in the future. Nevertheless, it should be mentioned that the establishment of a microbial biomarker is associated with several general problems, such as the dependence of the microbiome on ethnicity as well as geographic regions, and many others. In addition Zhang et al, have noted that although changes may have occurred in the oral microbiome of esophageal cancer patients they have observed inconsistencies in research over the years. This is particularly true for *Streptococcus*, which are widely distributed in both the mouth and esophagus. Consistency is disrupted by geographic locations or inconsistent laboratory methods for isolation and quantification.

The relevant conclusions about the changes of oral microbiota composition and levels from esophageal precancerous lesions to ESCC were drawn by Li et al. study (Li Z et al., 2021). The authors compared the saliva samples by PICRUSt2 analysis and showed that there are significant differences in terms of nitrate oxidoreductase alpha and beta subunits, and nitrate reductase gamma subunit activities between saliva of healthy subjects and patients with ESCC (Li Z et al., 2021). The role of these secondary metabolites produced by oral microbiota should be further investigated and their changes ought to be confirmed. Moreover, the authors proved that alpha diversity in the saliva decreased parallelly to disease progression (Li Z et al., 2021). They also identified common bacterial biomarkers in the group of patients with cancer: *Bosea*, *Solobacterium*, *Gemella*, and *Peptostreptococcus* and high – grade dysplasia: *Lactobacillus*. Considering that saliva samples are relatively easy to collect material, oral microbiota and for instance small RNAs (Li K et al., 2022), may be faster and efficient method of screening and diagnosis in the future and should be further investigated. The consistent results were also obtained in Wang et al. study which proved that alpha and beta diversity were significantly lower whereas the variability was higher comparing saliva of patients with ESCC to healthy subjects group (Wang et al., 2019). In addition, this study showed that high risk of esophageal cancer may be linked to both Actinomyces and Atopobium presence in the saliva (Wang et al., 2019). Although the investigated group is limited (20 patients with ESCC vs 21 healthy subjects), the conclusions are promising and should be tested in more numerous cohorts in the future.

**Figure 3:**
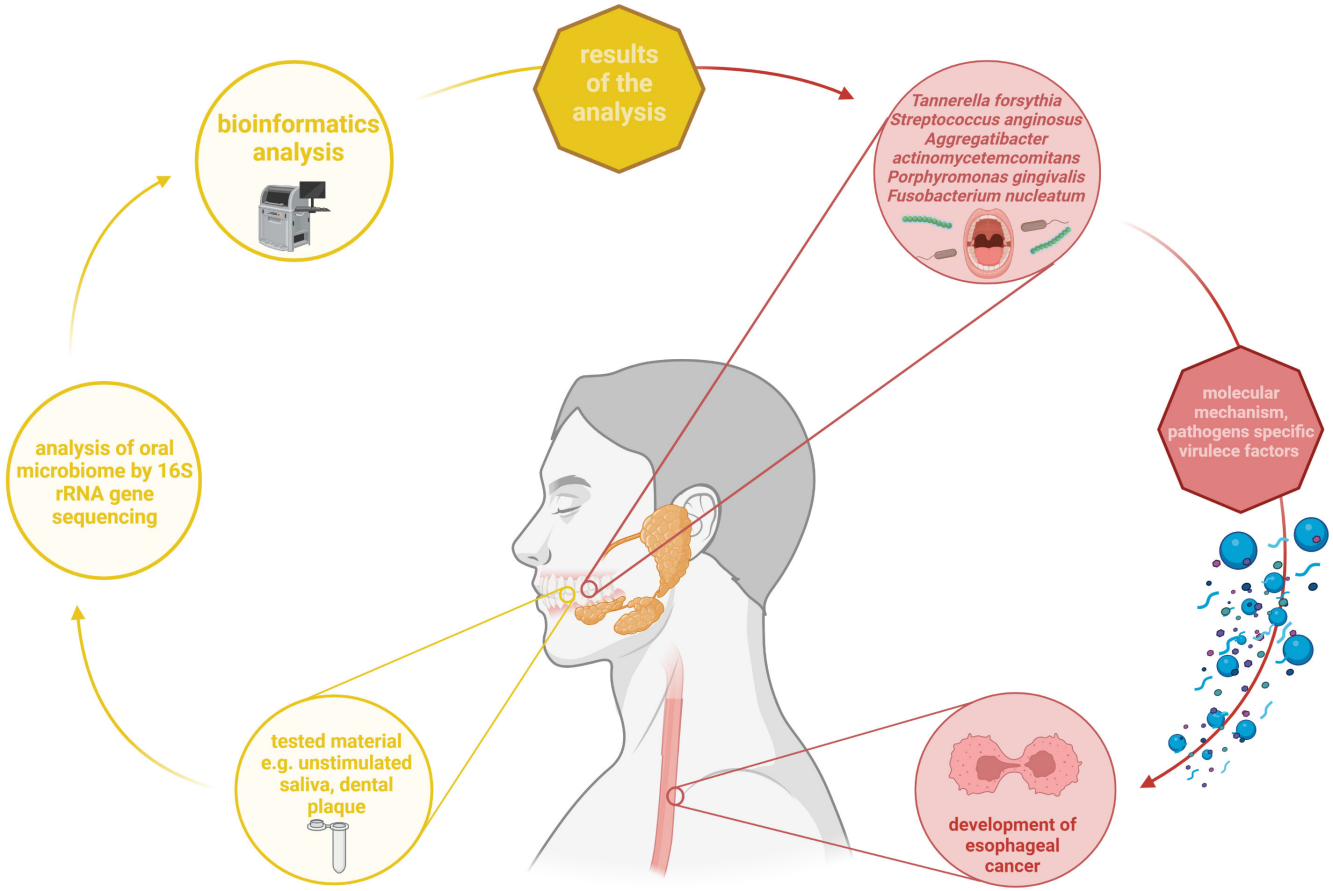
Summary of the relationship between changes in the oral microbiota and development of esophageal cancer. Based on up-to-date studies, some of bacteria such as *T. forsythia*, *S. anginosus*, *A. actinomycetemcomitans*, *P. gingivalis*, and *F. nucleatum* may contribute to esophageal cancer occurrence. They are characterized by presenting specific virulence factors that allow them to participate in this process. In most reviewed studies, oral microbiome was analysed from dental plaque and unstimulated saliva was analysed using 16S rRNA gene sequencing. Own elaboration based on the literature (Yamamura et al., 2016; Johansson et al., 2019; Chinthamani et al., 2021). This figure was created using *Biorender.com*.

Summarizing all above mentioned data/facts, it can be concluded that the link between alterations of oral microbiota and development of esophageal cancer exists. The summary of above mentioned aspects is presented on **Figure 3**.

## Consideration of probiotics as supportive treatment of esophageal cancer

There is observed increasing insight into the usage of probiotics in gastrointestinal cancers. Probiotics can be administered as supportive treatment of esophageal cancer to, among others, improve the nutritional status and functioning of immune system. As it was previously mentioned, it is estimated that 79% of esophageal cancer patients are malnourished (Jordan et al., 2018). Recently, in study protocol Liu et al. have reported that they will analyse the impact of probiotics on both nutritional status as well as gastrointestinal complications in patients with esophageal cancer in postoperative period (Liu et al., 2021). The impact of probiotics on nutritional status was analysed in Kaźmierczak-Siedlecka et al. double-blind, randomized and placebo-controlled study regarding cancer patients (also with esophageal cancer) (Kaźmierczak-Siedlecka et al., 2020). In this study probiotic strain *Lactobacillus plantarum* 299v was given per 4 weeks. It was observed that the level of albumin was significantly higher in group receiving probiotics (*p=0.032*). The level of albumin is one of the laboratory parameters used to assess the nutritional status besides prealbumin, total protein, and total lymphocyte count (Kaźmierczak-Siedlecka et al., 2020). Another probiotic *Lactobacillus rhamnosus* (PTCC 1637) may also be promising in case of esophageal cancer, what has been confirmed in Hashemi-Khah et al. study (Hashemi-Khah et al., 2022). Despite the fact that overall *Lactobacillus* spp. and *Bifidobacterium* spp. are the most commonly used probiotics (Kaźmierczak-Siedlecka et al., 2021), the other probiotics can also be considered. Nevertheless, specialists should pay attention to probiotic strains and their related properties.

## Conclusions

Changes that occur during the development of esophageal neoplasms affect not only the esophageal microbiota, but also other elements of the human body, including the oral cavity microbiome and the intestinal microbiome. The increasing amount of *F. nucleatum*, *S. anginosus* or *A. actinomycetemcomitans*, among others, during the development of the disease may serve as biomarkers in the future. However, further research is needed in this direction. Increased risk of esophageal cancer may be caused by improper diet or hygiene, leading to a proliferation of bacteria responsible not only for the development of esophageal cancer, but also for other diseases, including gingivitis or circulatory disorders. Last, but not least it should be mentioned that pathogens present in oral cavity such as *F. nucleatum* and *P. ginvivalis*, seem to have a strong influence on progression and final outcome. Therefore, new studies should be conducted to investigate the impact of possible eradication as a therapeutic option on survival and other prognostic parameters. Determining the most influential and discovering new prognostic microbes in the oral cavity should definitely be a goal for further studies.

## Author contributions

All authors listed have made a substantial, direct, and intellectual contribution to the work and approved it for publication.

## Conflict of interest

The authors declare that the research was conducted in the absence of any commercial or financial relationships that could be construed as a potential conflict of interest.

## Publisher’s note

All claims expressed in this article are solely those of the authors and do not necessarily represent those of their affiliated organizations, or those of the publisher, the editors and the reviewers. Any product that may be evaluated in this article, or claim that may be made by its manufacturer, is not guaranteed or endorsed by the publisher.
